# Effects of Parity and Serum Prolactin Levels on the Incidence and Regression of DMBA-Induced Tumors in OFA hr/hr Rats

**DOI:** 10.1155/2014/210424

**Published:** 2014-07-17

**Authors:** Corina V. Sasso, Flavia E. Santiano, Constanza M. López-Fontana, Virginia Pistone-Creydt, Marcelo E. Ezquer, María B. Hapon, Graciela A. Jahn, Rubén W. Carón

**Affiliations:** ^1^Laboratory of Hormones and Biology of Cancer, Institute of Medicine and Experimental Biology of Cuyo (IMBECU), CONICET, CCT-Mendoza, Avenida Adrián Ruiz-Leal s/n, CC855, 5500 Mendoza, Argentina; ^2^Institute of Sciences, Faculty of Medicine, German Clinique, University of Development, Santiago, Chile; ^3^Laboratory of Reproduction and Lactation, Institute of Medicine and Experimental Biology of Cuyo (IMBECU), CONICET, 5500 Mendoza, Argentina

## Abstract

Prolactin (PRL) is a key player in the development of mammary cancer. We studied the effects of parity or hyperprolactinemia on mammary carcinogenesis in OFA hr/hr treated with 7,12-dimethylbenzanthracene. They were divided into three groups: nulliparous (Null), primiparous (PL, after pregnancy and lactation), and hyperprolactinemic rats (I, implanted in the arcuate nucleus with 17*β*-estradiol). The tumor incidence was similar in the three groups. However, a higher percentage of regressing tumors was evident in the PL group. Serum PRL, mammary development, and mammary *β*-casein content were higher in I rats compared to Null. The expression of hormone receptors was similar in the different groups. However, mammary tissue from PL rats bearing tumors had increased expression of PRL and estrogen alpha receptors compared to rats free of tumors. Our results suggest that serum PRL levels do not have relevance on the incidence of tumors, probably because the low levels of PRL in OFA rats are not further decreased by PL like in other strains. However, supraphysiological levels of PRL affect carcinogenesis. PL induces regression of the tumors due to the differentiation produced on the mammary cells. Alterations in the expression of hormonal receptors may be involved in progression and regression of tumors.

## 1. Introduction

Parity is an effective protective factor against breast cancer in women and provides protection against chemically induced mammary carcinogenesis in rats [1–5]. Although rodent experimental data and human epidemiological evidence consistently show a protective effect of pregnancy on mammary carcinogenesis, the mechanisms underlying this protection are still unclear [6]. The pregnancy-associated refractoriness of the mammary gland to carcinogenesis is caused in part by lasting phenotypic alterations of the mammary epithelia that occur during pregnancy and lactation [7]. The endocrine milieu is also a determining factor in the parity-associated refractoriness to mammary carcinogenesis. It has been shown that treatment of rats with both estrogen (E_2_) and progesterone (P_4_) for a short period of time protects against mammary carcinogenesis [5, 8].

With some controversy prolactin (PRL) has been proposed as a key player in the development of mammary cancer in rodent models. Thus, a direct correlation between serum PRL levels and susceptibility of various rat strains to induction of mammary tumors by chemical carcinogens has been suggested [9]. There is also a direct correlation between drug-induced hyperprolactinemia and increased tumor growth and between hypoprolactinemia and retarded tumor growth [10].

The OFA hr/hr rats are hairless rats derived from Sprague-Dawley with a genetic deficient lactation caused by an impaired response to the suckling stimulus and heightened susceptibility to stress [11, 12]. The nature of the mutation has been shown to be a large intragenic deletion of the desmoglein-4 gene (Dsg-4) encompassing nine exons [13–15], which codifies for a protein belonging to the desmoglein family. The products of these genes, expressed in neural and neuroendocrine tissues, are cell-adhesion molecules related to cadherins. Similarly to the effects of parity on women, the OFA rats have a persistent reduction in the concentration of serum PRL and blunted PRL response to secretagogues.

To assess the involvement of serum PRL in the susceptibility of the mammary gland to carcinogenesis, we used OFA hr/hr rats and we compared the effects of parity and lactation or induced hyperprolactinemia on mammary carcinogenesis. In the present study, we show that the incidence of mammary tumors induced by DMBA in OFA rats is not influenced by serum PRL and that the progression and regression of the tumors are dependent on the extent of differentiation produced in the mammary gland by the effects of parity and lactation.

## 2. Materials and Methods

### 2.1. Animals

Virgin OFA hr/hr female rats (180–200 g) bred in our laboratory were used. The animals were kept in a light (lights on 06.00–20.00 h) and temperature (22–24°C) controlled room; rat chow (Cargill, Córdoba, Argentina) and tap water were available* ad libitum*.

Animal maintenance and handling were performed according to the NIH guide for the Care and Use of Laboratory Animals (NIH publication number 86-23, revised 1985 and 1991) and the UK requirements for ethics of animal experimentation (Animals Scientific Procedures, Act 1986).

### 2.2. Experimental Design

All rats were treated* per os* with a single dose (15 mg/rat) of 7,12-dimethylbenzanthracene (DMBA, Sigma, Buenos Aires) at 54–56 days of age and they were included at random in one of the three experimental groups:

- controls (nulliparous rats -Null- *n* = 42): rats exposed to DMBA without subsequent treatment;
- pregnancy and lactation (primiparous rats -PL-, *n* = 82): twenty-five days after carcinogen administration (80 days of age), rats were caged with fertile males on proestrus afternoon. The following day, all the rats showing spermatozoa in their vaginal smears were caged individually and they were checked for the occurrence of mammary tumor twice a week during the 21 days of pregnancy and afterward for 20 days of lactation. Twenty-four hours after delivery, all the litters were adjusted at 8 pups and were maintained with their mothers until weaning. As previously published [12], OFA rats have normal fertility but 50% of the litters die of malnutrition on early lactation; only 6% of the mothers show normal lactation. Thus, all pups without signs of being properly nourished were replaced for age-matched pups. This procedure did not influence the incidence and the proportion of tumor in regression;
- estradiol implants (hyperprolactinemic rats -I-, *n* = 37): twenty-five days after carcinogen administration, the rats were implanted in the arcuate nucleus with 17*β*-estradiol as previously described [16–18]. Briefly, rats were anesthetized by i.p. injection of ketamine hydrochloride (40 mg/kg, i.p.) and xylazine (8 mg/kg, i.m.) and placed in a stereotaxic frame. In order to access the area immediately above the medial PeV-ARC region, stainless steel cannulae (ID, 0.33 mm; OD, 1.78 mm; Small Parts, Miami, FLA) were bilaterally implanted through burr holes drilled through the skull over the target sites. The tip of the cannulae was brought to the following coordinates relative to the bregma: 3.0 mm posterior, 8.5 mm ventral, and 0.6 mm right and left. Each cannula was filled with approximately 0.5 ± 0.05 mg of crystalline 17*β*-estradiol (Sigma Chem. Co., St. Louis, MO). The upper end of cannulae protruded 2-3 mm from the skull and was fixed to the bone with dental acrylic cement (Subiton, Surrey, UK). The accuracy of cannula placement was checked after euthanasia in the fixed brains by identification of the needle track in brain sections. In general, the tips of the cannulae were above the ARC-PeV region. In previous studies, we have demonstrated that the implant of empty cannulas (or filled with placebo) into the arcuate nucleus has no significant effects on PRL secretion [16–18].

All animals were palpated twice a week, starting at day 30 after DMBA administration and for at least 200 days for tumor detection. Incidence was calculated as the percentage of rats that had tumors respect to the total number of rats per group. The rats were decapitated between 10.00 and 12.00 h on the day the tumors reached a tumor volume >1000 mm^3^ or at the end of the experiment on day 200 when they did not develop mammary tumors. Trunk blood samples were allowed to clot at room temperature. Serum was stored at −20°C until assayed for hormones determinations. After decapitation, a piece of normal mammary gland and the tumors were removed for *β*-casein content and histopathological analysis.

### 2.3. Hormone Assays

PRL and GH were measured by a double-antibody RIA as previously described [19] using materials kindly provided by Dr. A. F. Parlow and the NHPP (National Hormone and Pituitary Program, Harbor-UCLA Medical Center, Torrance, CA, USA). Hormones were radioiodinated using the chloramine-T method. Results are expressed in terms of the rat PRL RP-3 and GH RP-2 standard preparations. Assay sensitivity was 0.5 *μ*g/L and the inter- and intra-assay coefficients of variation were less than 10% for both hormones.

### 2.4. *β*-Casein Determination

Mammary *β*-casein was measured as previously described [20, 21]. Briefly, 200 mg of mammary tissue was cut into small pieces and homogenized in 2 mL 50 mM sodium phosphate buffer, 150 mM NaCl, 0.1% NaN_3_, 0.1% Triton X-100, pH 7.6 with an Ultraturrax homogenizer. The homogenates were centrifuged at 600 g for 30 min. The supernatants were used for *β*-casein determination by a homologous radioimmunoassay according to Edery et al. [22] as modified in our laboratory [23]. All samples were assayed in duplicate. The standard curve of rat *β*-casein was between 0.25 and 512 ng/mL and the sample values were calculated per mg of tissue.

### 2.5. Tumor and Mammary Gland Histology

A small piece of tumor and inguinal mammary gland (contralateral to the tumor) from each rat were processed for histopathologic studies. Sections of 3–5 *μ*m thickness were cut with a microtome and stained with hematoxylin-eosin (H&E) to define the histopathological changes in the mammary glands and to classify tumors according to published criteria [24, 25]. Images were taken with a Nikon Eclipse E200 Microscope fitted with a digital still camera Micrometric SE Premium (Nikon Corp., Japan) under 100x, 400x, and 600x magnifications. The quantification of the percentages of stroma, mostly composed by adipocytes and epithelial tissue in the mammary gland, was performed by measuring the area occupied in 8–10 fields of each preparation from all rats using the ImageJ 1.42q software available at the NIH site (http://rsb.info.nih.gov/ij). Each area was expressed as a percentage of the whole field as previously published [25, 26].

### 2.6. RNA Isolation and RT-PCR Analysis

Total RNA from normal mammary glands and tumors was extracted using the Chomczynski-Sacchi method modified by Puissant and Houdebine [27] to determine the expression of the following hormone receptors: E_2_ receptor *α* (ER*α*), E_2_ receptor *β* (ER*β*), PRL receptor (PRLR), P_4_ receptor (PR), and GH receptor (GHR). Ten micrograms of total RNA were reverse transcribed at 42°C using random hexamer primers and Moloney murine leukemia virus RT (Invitrogen/Life Technologies, Buenos Aires, Argentina) in a 20 *μ*L reaction mixture. Before proceeding with the semiquantitative PCR, the conditions were established for each tissue such that the amplification of the products was in the exponential phase, and the assay was linear with respect to the amount of input RNA.

All reactions were carried out for 30 cycles, with the following cyclic parameters, 95°C for 1 min, 62°C for 1 min, and 72°C for 2 min, and then terminated with a 5 min extension at 72°C. RNA samples were assayed for DNA contamination by PCR without prior reverse transcription. The PCR products were analyzed on 1.5% agarose gels containing 0.5 mg/mL ethidium bromide and photographed with a Polaroid camera. Band intensities of RT-PCR products were quantified using the ImageJ 1.42q software available at the NIH site (http://rsb.info.nih.gov/ij). Relative levels of mRNA were expressed as the ratio of signal intensity for the target genes relative to that for *β*-actin. The sequence of the primers used for each gene and the size of the product of amplification obtained are shown in Table 1.

### 2.7. Statistical Analysis

Values are given as means ± S.E.M. of 11–47 animals per group. All statistical analysis was performed using GraphPad Prism 5.01 software (GraphPad Software Inc., CA, USA). Differences in the distribution of variables between the three studied groups were assessed using oneway analysis of variance (ANOVA I) or Kruskal-Wallis test depending on the normality of the variables as evaluated by the Kolmogorov-Smirnov test. Two-way analysis of variance (ANOVA II) was used for analysis of differences between rats with and without mammary tumors in the three groups. Post hoc comparisons between means were made by Bonferroni's test or Dunn's Multiple Comparison test. Student's* t*-test was used when only two groups were compared. When variances were not homogeneous, logarithmic transformation of data was applied. Incidence and percentages of mammary epithelial areas were analyzed by chi-square. Differences were considered significant if the probability was 5% or less.

## 3. Results

### 3.1. Incidence, Progression, and Regression of Mammary Tumors

Tumor incidence (Table 2), multiplicity, and latency (not shown) were not statistically different among the three groups. However, a nonsignificant tendency to lower values was observed in the PL rats. Interestingly, a significant rise (*P* < 0.001) in the percentage of tumors with regression and with macroscopic signs of necrosis was evident in the PL rats (Table 2).

### 3.2. Serum Prolactin and GH Levels

**Figure 1:**
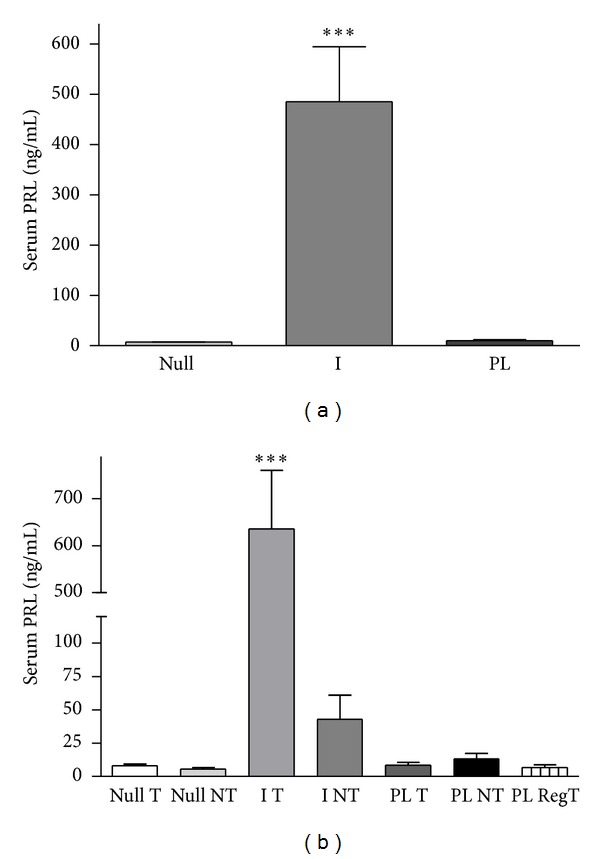
Serum prolactin (PRL) levels in DMBA-treated rats at the end of the experiment. (a) Nulliparous rats (Null) and rats after a cycle of pregnancy and lactation (PL) had significantly lower serum PRL than rats implanted with 17-*β* estradiol in the arcuate nucleus (I) (*P* < 0.0001). This group is referred to as hyperprolactinemic rats. (b) When classified depending the development (T), absence (NT), or regression (PL RegT) of mammary tumors, the implanted rats developing tumors showed significantly higher levels of serum PRL than the rest of the animals (*P* < 0.0001) including implanted rats without tumors.

As expected, serum PRL concentration at the end of the experiment was significantly higher in I rats (*P* < 0.0001) compared to Null or PL (Figure 1(a)). No correlation between serum PRL levels at the end of the experiment and percentages of regression of tumors was found.

No significant differences in serum PRL levels were observed in Null or PL rats that developed mammary tumors from those that did not. However, a statistically significant increase (*P* < 0.01) was found in I rats that bore mammary tumors compared to those that did not (Figure 1(b)).

Circulating GH levels were similar in all the groups regardless of tumor development (results not shown).

### 3.3. Mammary Gland Tissue Differentiation and Development

**Figure 2:**
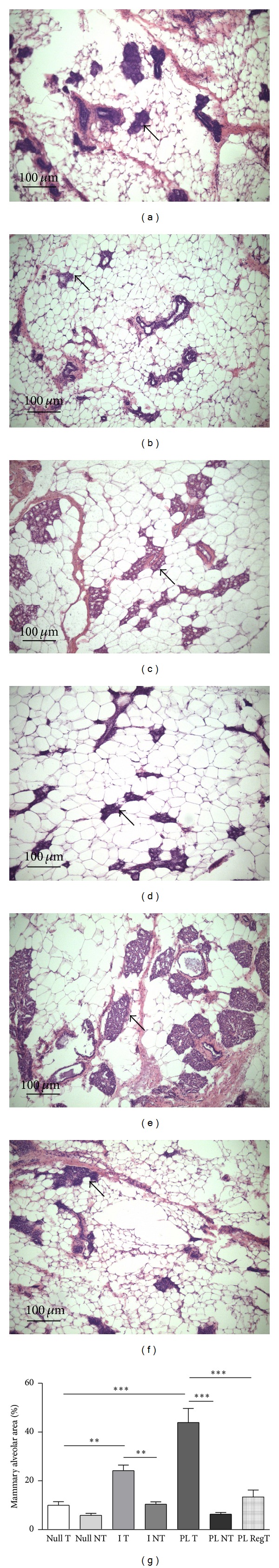
Hyperprolactinemia or pregnancy and lactation modify the ratio parenchyma/stroma in the mammary gland. Representative microphotographs (100x) of H&E-stained normal mammary tissue from nulliparous rats ((a) and (b)), hyperprolactinemic rats ((c) and (d)), and primiparous rats ((e) and (f)), with development ((a), (c), and (e)), or absence ((b), (d), and (f)) of mammary tumors. (g) Quantifications of the relative percentages of the alveolar area. Values represent mean ± S.E.M. of 8–10 fields of each preparation from 11–26 animals per group. **P < 0.001, ***P < 0.0001 comparing the selected groups. Comparisons were performed by ANOVA I. Arrows show alveolar structures.

The effect of pregnancy and partial lactation on the development of the mammary gland was evaluated by histological observation and measurement of the areas occupied by parenchyma or stroma.  Figure 2 shows representative microphotographs of H&E-stained mammary tissue from Null rats ((a) and (b)), I rats ((c) and (d)), and PL rats ((e) and (f)). The mammary glands of Null rats had a normal appearance with few ducts surrounded by a small amount of fibrous connective tissue and abundant adipocytes. A similar image was seen in hyperprolactinemic rats but with a higher development of alveolar structures. The mammary tissue from primiparous rats showed a more noticeable development of lobuloalveoli.  Figure 2(g) shows that the percentages of alveolar tissue in mammary glands from rats that bore tumors were higher than in those rats that did not develop cancer, particularly evident in I rats (*P* < 0.01) and in PL rats (*P* < 0.001).

### 3.4. Mammary Content of *β*-Casein

**Figure 3:**
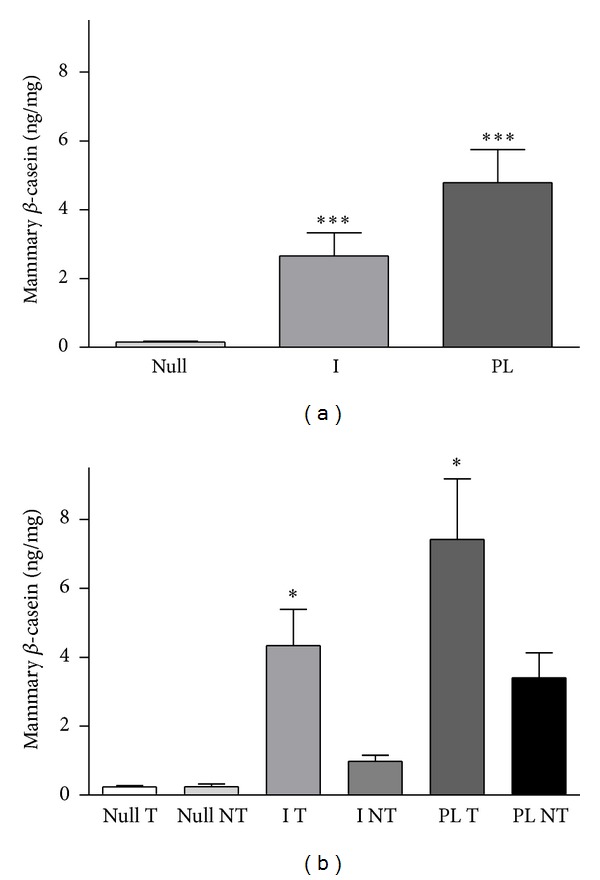
Mammary *β*-casein content at the end of the experiment from DMBA-treated rats. (a) Nulliparous rats (Null) had significantly lower mammary *β*-casein than rats implanted with 17-*β* estradiol in the arcuate nucleus (I) or rats after a cycle of pregnancy and lactation (PL) ***P < 0.0001. (b) When classified depending on the development (T) or absence (NT) of mammary tumors, the implanted and primiparous rats developing tumors showed significantly higher levels of mammary *β*-casein than the corresponding tumor-free rats *P < 0.05. Comparisons were performed by ANOVA I.

As expected, the mammary content of *β*-casein, an indicative of the degree of differentiation, was increased (*P* < 0.001) in I rats compared to Null rats. A significant increase in mammary *β*-casein was produced in PL rats (*P* < 0.001, Figure 3(a)) even long after the pregnancy lactation cycle. Moreover, when the rats were grouped according to the development of tumors, no significant differences in mammary *β*-casein were observed in Null rats with and without mammary tumors. However, I rats or PL rats had higher levels of *β*-casein (*P* < 0.05 and *P* < 0.001, resp.) if they had developed mammary tumors (Figure 3(b)).

### 3.5. Expression of Hormone Receptors in the Nontumoral Mammary Tissue

**Figure 4:**
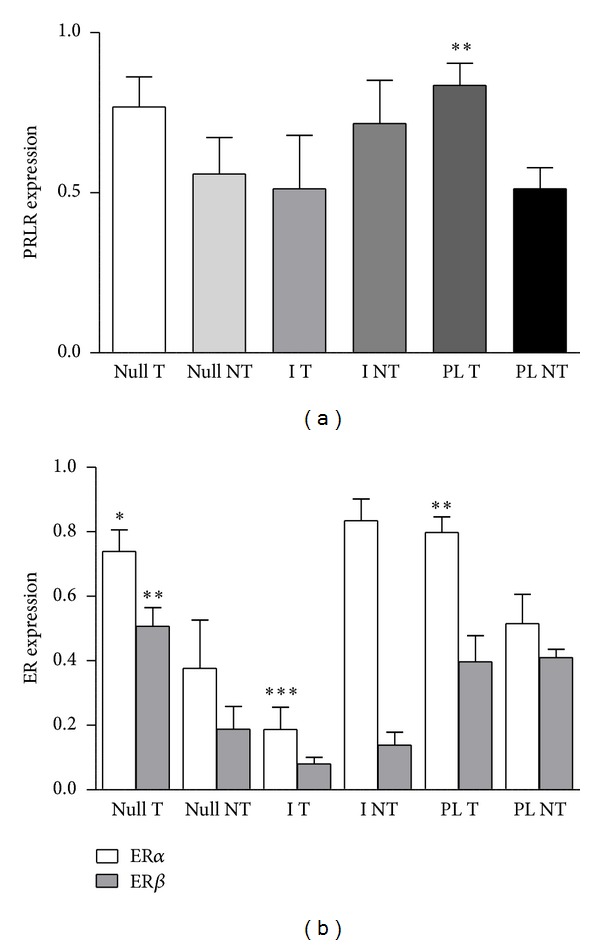
Expression of mRNA for hormones receptors in mammary glands from DMBA-treated rats at the end of the experiment. (a) Expression of mRNA for PRLR relative to *β*-actin. **P < 0.01 with respect to the corresponding tumor-free group. (b) Expression of mRNA for ER*α* and *β* relative to *β*-actin. *P < 0.05, **P < 0.01, ***P < 0.0001 with respect to the corresponding tumor-free groups. Comparisons were performed by ANOVA I.

We next investigated the level of expression of hormone receptors in the normal tissue of the mammary gland from the three groups. The expression of PRLR was similar in the three groups. However, mammary tissue from PL rats bearing tumors had increased (*P* < 0.01) content of PRLR mRNA compared to rats free of tumors (Figure 4(a)). No difference in the expression of total PR mRNA was found between the three groups of treatments irrespective of the development of tumors (not shown).

The overall expression of ER*α* in the mammary gland of I rats was similar to that of Null or PL rats (data not shown). However, the mammary gland of I rats that had developed tumors had a diminished expression of ER*α* in comparison to Null or PL rats bearing mammary tumors (*P* < 0.001, Figure 4(b)). On the contrary, the expression of ER*α* was increased in the mammary gland of tumor-bearing Null or PL rats compared to the respective tumor-free rats (*P* < 0.05 and *P* < 0.01, respectively, Figure 4(b)).

No significant differences in the expression of ER*β* were observed between mammary glands from Null, I, or PL rats (results not shown). However grouping the rats according to the presence or not of tumors showed a higher expression of ER*β* only in the Null group (*P* < 0.01, Figure 4(b)).

### 3.6. Changes Related to the Pregnancy and Lactation Cycle in the Mammary Tumors

**Figure 5:**
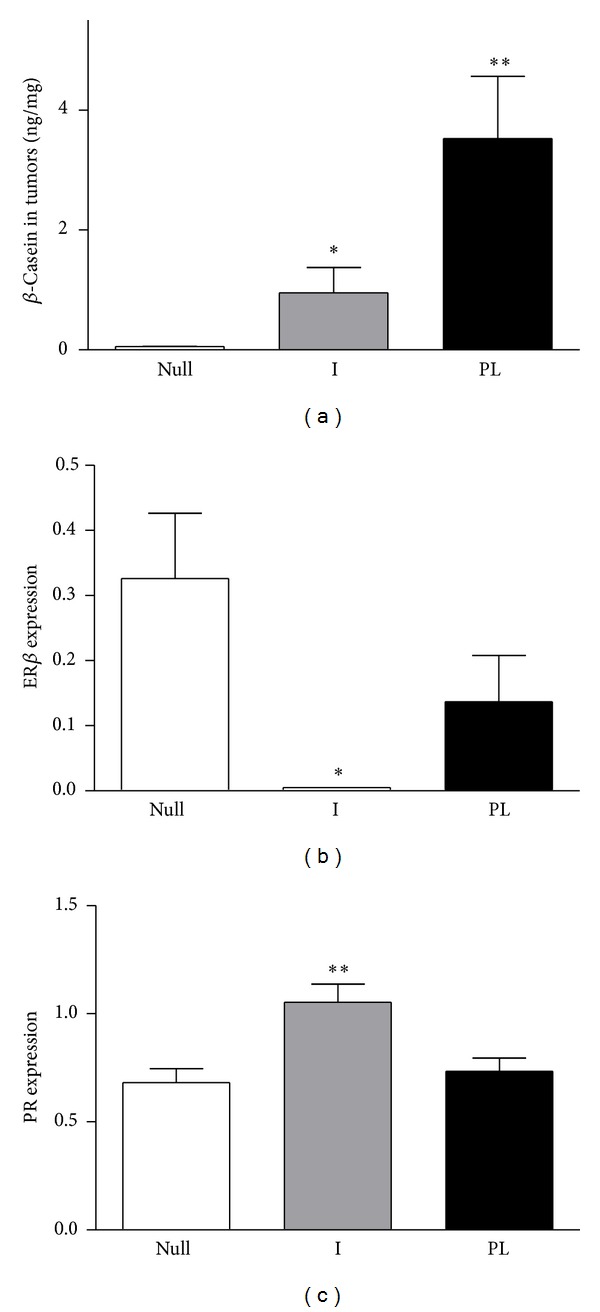
*β*-Casein content and expression of mRNA for hormones receptors in tumors from DMBA-treated rats. (a) Tumor tissue from nulliparous rats (Null) contains significantly lower amounts of *β*-casein than tumors from implanted (I) or primiparous (PL) rats (*P* < 0.05 and *P* < 0.001, resp.). (b) The expression of mRNA of ER*β* is lower in the tumors of I rats than in the other two groups (*P* < 0.05). (c) mRNA expression of PR is significantly higher in I rats with respect to Null or PL rats (*P* < 0.01).

We compared the expression of *β*-casein in the tumors from the three groups to determine the influence of the different physiological backgrounds on the changes related to tumor transformation. The mammary tumors from PL rats produced significantly higher levels of *β*-casein than the Null or I rats (Figure 5(a)). Moreover, 23.4% of the tumors from primiparous rats showed macroscopic and microscopic evidence of necrosis (Table 2).

Both PRLR and ER*α* expressions were similar in tumors developed in Null, PL, or I rats (results not shown). However, tumors from I rats showed undetected expression of ER*β* and an increased expression of PR (Figures 5(b) and 5(c)).

### 3.7. Changes in the Mammary Gland Related to the Regression of Tumors

**Figure 6:**
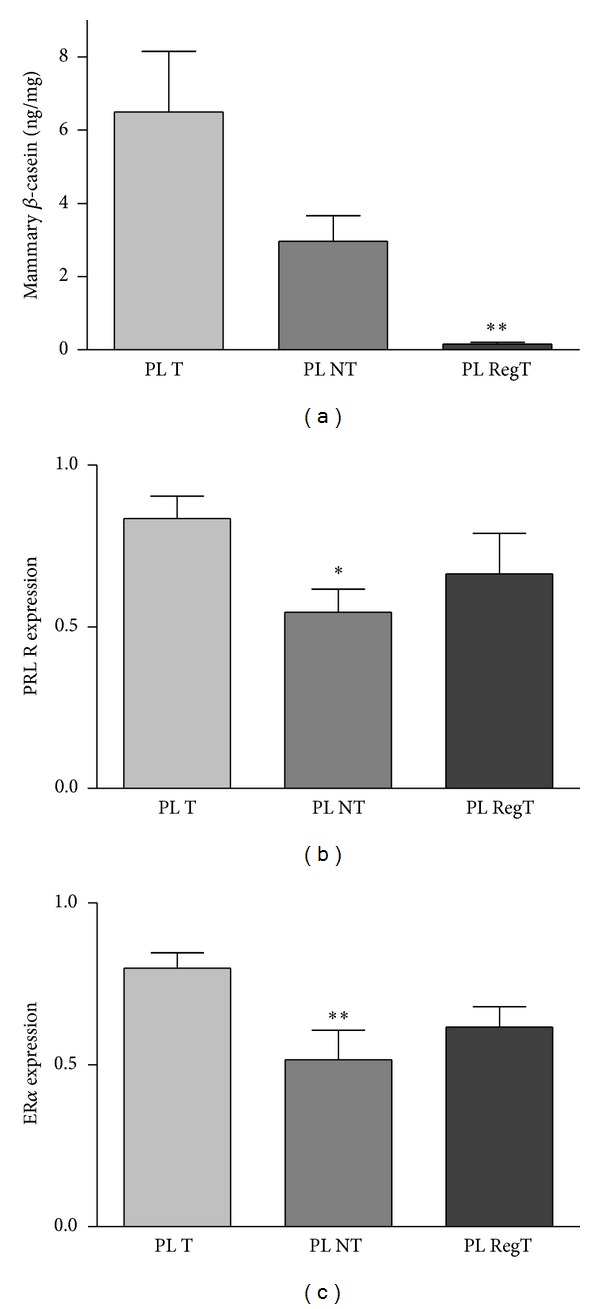
*β*-Casein content and expression of mRNA for hormones receptors in mammary glands from primiparous rats (PL). Mammary *β*-casein content was lower (*P* < 0.01) in mammary glands from PL rats that had tumor regression (PLRegT) than in those that did not have tumors (PLNT) or did have no regressing tumors (PLT). (b) and (c) PRLR and ER*α* expressions were lower (*P* < 0.05 and *P* < 0.01, resp.) in mammary glands from tumor-free PL rats compared to the other two groups.

In order to investigate whether the increased percentage of regressing tumors in the group of primiparous rats is due to long lasting changes in the hormonal milieu or to the grade of differentiation of the mammary gland, we compared primiparous rats bearing tumors with those without tumors and rats with regressing tumors. No significant differences were found between serum PRL concentrations from the PL rats that did not develop tumors, those that bore nonregressed tumors, and those that bore regressed tumors (not shown). However a decrease (*P* < 0.01) in the mammary content of *β*-casein was evident in the group of rats that developed mammary tumors and had tumor regression (Figure 6(a)), suggesting a link between regression of the tumors and involution of the mammary gland.

PRLR expression was significantly lower in mammary glands from rats that did not develop tumors, but no different from those that had regression of the tumors (Figure 6(b)). A similar decrease was observed in the expression of ER*α* (Figure 6(c)). No significant differences were found in mammary expression of PR or ER*β* (results not shown) between the three groups.

**Figure 7:**
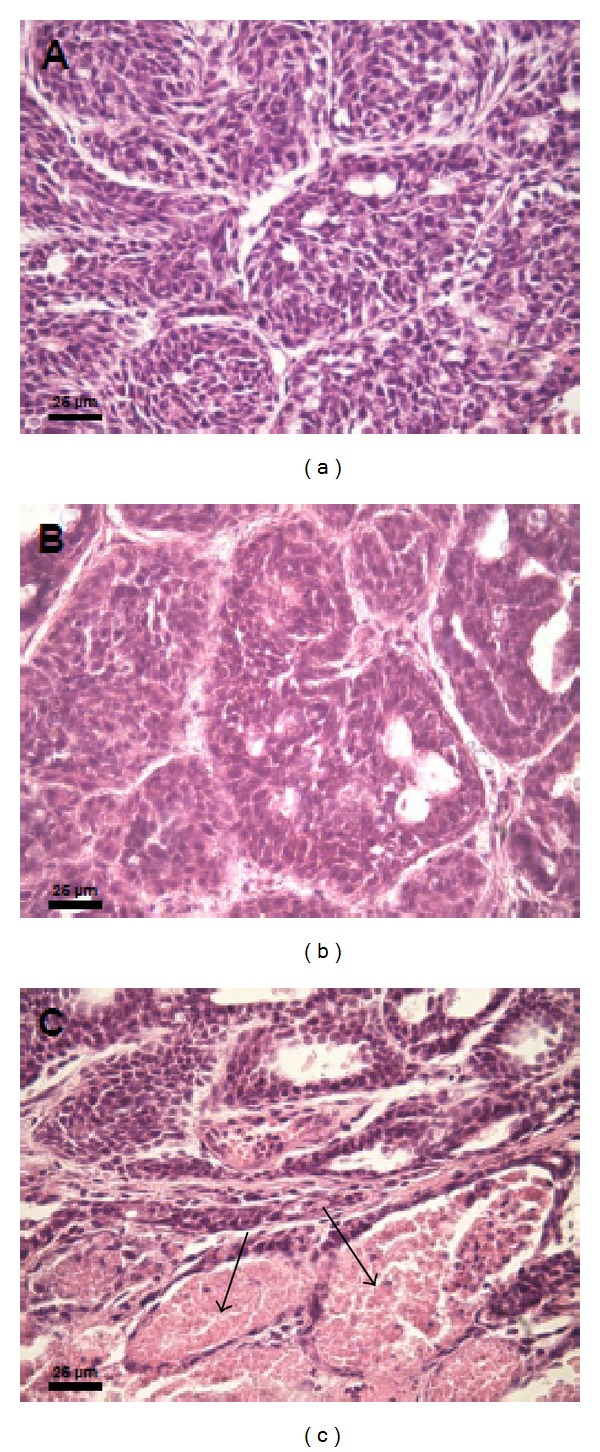
Representative microphotographs (100x) of H&E-stained normal tumors from nulliparous rats (a), hyperprolactinemic rats (b), and primiparous rats showing a noticeable area of necrosis (c). Arrows show necrotic areas.

As shown in Table 2, more than 20% of the tumors from PL rats showed evident areas of necrosis.  Figure 7 shows three representative microphotographs stained with H&E of tumor from Null, I, and PL rats, which shows necrosis.

## 4. Discussion

To study the involvement of lactogenic hormones in mammary carcinogenesis, we compared our Null or PL rats with virgin rats made hyperprolactinemic through hypothalamic implants of estradiol. In this model, serum estradiol remains at a physiological level [16–18] allowing us to investigate the effects of PRL excluding the well-known actions of high amounts of E_2_ on mammary carcinogenesis.

A correlation between serum PRL levels and susceptibility of various rat strains to chemically induced mammary carcinogenesis has been suggested [9]. Both GH and PRL have been previously shown to be reduced after parturition as compared with nulliparous, age-matched animals [28]. Moreover, parous rats showing almost complete refractoriness to chemical carcinogenesis acquire high susceptibility after hormonal treatment that increases serum GH and PRL levels [29]. In our OFA rats, neither serum GH nor circulating PRL decreased after PL. Accordingly, even when the incidence of mammary tumors was slightly lower in primiparous rats, the difference respect to nulliparae was not statistically significant, suggesting that the cycle of pregnancy/lactation failed to prevent tumor development in OFA hr/hr rats. This different behavior from the original strain (Sprague-Dawley) [28, 29] regarding the lack of protection induced by pregnancy and lactation might be due to the lower levels of circulating PRL during lactation in the PL OFA rats. Thus, the deficient lactation may have been insufficient to protect the mammary tissue from tumoral transformation. Moreover, despite the persistent hyperprolactinaemia produced in I rats, the incidence of tumors was similar to the other groups, suggesting a null impact of serum PRL levels on mammary carcinogenesis. Interestingly, in I rats, serum PRL was significantly higher in those rats that developed tumors, suggesting that supraphysiological levels of PRL may be relevant in increasing the risk of mammary cancer.

Our results support the idea that the mammary development* per se* is not enough to account for the protective effect of parity. In fact, the implant of estradiol in the arcuate nucleus produces hyperprolactinemia and luteal phase with high levels of circulating P_4_ and mammary development and differentiation similar to pregnancy [16, 17]. Thus, a protective effect of the implants dependent on the differentiation of the mammary gland could be expected. However, the incidence of mammary tumors in the I group was similar to that in the Null rats, suggesting that the sustained hyperprolactinaemia is not enough to increase carcinogenesis and, moreover, the development of the mammary gland achieved in the implanted rats is not enough to prevent chemical carcinogenesis. In agreement with our results, chronic treatment with perphenazine, producing hyperprolactinemia and high levels of P_4_, did not protect the mammary gland against chemical induction of tumors [30].

It is worth noting that, in our model, mammary *β*-casein content paralleled the alveolar area measured in the histological preparations, suggesting that the proliferation of epithelial tissue of the mammary gland is accompanied of certain differentiation. This result is in agreement with a previous study in normal Wistar rats [26].

Interestingly, the percentage of mammary tumors in regression was greatly affected by PL, indicating that the classical protective effect of gestation described by others may be due, at least in part, to an increased number of tumors undergoing regression rather than a decrease of the development of mammary tumors. Our overall results suggest that a cycle of PL has “toxic” effects on the mammary tumors, which cannot be explained only by the milieu of mammogenic hormones, since we did not find any statistically significant difference on serum GH (results not shown) or PRL values, between nulliparous or primiparous rats. On the contrary, the significantly lower content of *β*-casein in the mammary gland from rats that had regression of their tumors constitutes an interesting support of the idea that involution of the normal epithelia can be implied in the regression of tumors, especially so, when the tumors of the rats with pregnancy and lactation retain the capability to synthesize *β*-casein. Studies currently under way in our laboratory are focusing on the expression of several molecular markers involved in the process of involution of the mammary gland after lactation in the regressing tumors.

We did not find correlation between expression of ARNm of PRLR and serum PRL levels. However, mammary glands from PL rats that had developed tumors had increased expression of PRLR compared to PL rats free of tumors. This difference can explain the higher alveolar development reached in those rats even when the values of circulating PRL at the moment of sacrifice were similar in both groups. The increased development of alveolar tissue may cause, in turn, increased content of *β*-casein. It is worth to note that both I and PL rats had augmented alveolar development compared to Null rats, but the level of differentiation in terms of *β*-casein expression is higher in PL rats, supporting a role for the extent of differentiation more than the alveolar development in the protective effect of PL.

To assess whether the transformation of the mammary cells involves changes in the expression of receptors we studied in parallel the tumors and tissue from the contralateral normal mammary gland in the same animals. The expression of ER*α* increased in tumors of hyperprolactinemic rats compared to the contralateral mammary gland, but it was unmodified in Null or PL rats (results not-shown). The ability of PRL to stimulate the expression of both types of ER has been shown in the rat corpus luteum, in the mammary gland, and in the decidua (see [31] for a review). Previous reports suggest that PRL can stimulate ER expression in some breast cancers [32, 33]. Moreover, PRL can activate ER*α* even in the absence of estrogenic ligands leading to oncogenesis [34]. In our study, the expression of ER*β* decreased in the tumors from the three groups compared to their respective mammary glands (not shown), suggesting a protective role for ER*β*. This result supports previous studies showing a decrease in the expression of ER*β* in the process of human breast cancer progression associated with poor differentiation [35]. Moreover, the expression of ER*α* was higher in untransformed mammary tissue from Null or PL rats that developed mammary tumors compared with those that did not. On the contrary, hyperprolactinemic rats that developed tumors had lower expression of ER*α* in their mammary tissue. This particular result may suggest that when supraphysiological levels of circulating PRL are constantly acting on the mammary gland, tumors can progress even with lower expression of ER*α*.

In conclusion, in our model of carcinogenesis using OFA hr/hr rats we showed that serum PRL levels do not seem to have relevance on the incidence of tumors, probably because the relative low levels of PRL in those rats are not further decreased by pregnancy and lactation like in other strains. Even though, supraphysiological levels of serum PRL may affect carcinogenesis.

On the other hand, PL induces regression of the tumors most probably due to the degree of differentiation produced on the mammary cells than to the extent of proliferation reached by the epithelial tissue. Alterations in the expression of hormonal receptors may be involved in both progression and regression of the tumors.

## Figures and Tables

**Table 1:** Sequence of primers used in the respective PCR and molecular size of the amplification product.

RNAm	Sense 5′-3′	Antisense 3′-5′	Size
Actin	CGTGGGCCGCCCTAGGCACCA	TTGGCCTTAGGGTTCAGAGGGG	243
ER*α*	AATTCTGACAATCGACGCCAG	GTGCTTCAACATTCTCCCTCCTC	345
ER*β*	AAAGCCAAGAGAAACGGTGGGCAT	GCCAATCATGTGCACCAGTTCCTT	204
PRLR	AAAGTATCTTGTCCAGACTCGCTG	AGCAGTTCTTCAGACTTGCCCTT	279
PR	CCCACAGGAGTTTGTCAAGCTC	TAACTTCAGACATCATTTCCGG	325
GHR	GAGGAGGTGAACACCATCTTGGGC	ACCACCTGCTGGTGTAATGTC	534

**Table 2:** Number and percentages of rats with and without mammary tumors in the three groups.

	Without tumors	With tumors	%	Regressed tumors	%	Necrotic tumors	%	Regular tumors	%
Nulliparous	12	30	71.4	0	0	0	0	30	100
Pregnancy-lactation	35	47	57.3	17	36.2∗	11	23.4∗	19	40.4
Implanted	11	26	70.3	0	0	0	0	26	100

∗ *P* < 0.05 compared to Nulliparous or Implanted rats.
